# Supplementary material for: The role of financial stress, food insecurity, and COVID-19-related illness concerns shaping mental health in five South Asian countries during the pandemic (2020–2022): A secondary analysis of the online COVID-19 Trends and Impact Survey (CTIS) data
Source: PLOS Glob Public Health. 2025 Aug 8;5(8):e0004704. doi: 10.1371/journal.pgph.0004704 (PMC12334018; doi:10.1371/journal.pgph.0004704)
Supplement: S1 Text — Data processing for demographic variables. (PDF) [file pgph.0004704.s001.pdf]

S1 Text

Gender: Gender was self-reported and categorized as “male,” “female,” “other,” and “prefer not to answer.” For analysis, gender was treated as a binary variable, excluding participants who selected “other,” (N = 2,453; 0.10% of participants with valid responses) and “prefer not to answer” (N = 16,676; 0.70% of participants with valid responses).

Age: Participants initially self-reported their age in the following categories: 18–24, 25–34, 35–44, 45–54, 55–64, 65–74, and 75+ years at the time of response. Due to sample size limitations, ages 65–74 and 75+ were combined into a single category (65+ years). Additionally, to align with benchmark data, we combined ages 25–34, 35–44, 45–54, and 55–64 into a broader 25–64 years category.

Education: In Period 1, participants reported years of education completed as an open-ended response. In Period 2, this was revised to a multiple-choice question on the highest level of education attained. To reduce measurement error, responses from Period 1 were limited to non-negative values below 50 years of education. Education data from both periods were dichotomized into two categories: less than a high school diploma (less than 12 years; coded as 0) and high school diploma or higher (12 years or more; coded as 1).

Residential status: Residential settings were converted into a binary variable: rural (village or rural; coded as 0) and urban (city or town; coded as 1).

Occupation: Occupation was analyzed as a categorical variable, grouping participants into broad categories: agriculture, construction, education, health, tourism, transportation, and “other.” The “other” category included occupations such as electricity/water/gas/waste, financial/insurance/real estate services, manufacturing, mining, personal services, professional/scientific/technical activities, public administration, and unspecified responses.

Vaccination status: Vaccination status was analyzed as a binary variable: “not vaccinated” (coded as 0 for “no” or “I don’t know”) and “vaccinated” (coded as 1 for “yes”).
