# Supplementary material for: The role of financial stress, food insecurity, and COVID-19-related illness concerns shaping mental health in five South Asian countries during the pandemic (2020–2022): A secondary analysis of the online COVID-19 Trends and Impact Survey (CTIS) data
Source: PLOS Glob Public Health. 2025 Aug 8;5(8):e0004704. doi: 10.1371/journal.pgph.0004704 (PMC12334018; doi:10.1371/journal.pgph.0004704)
Supplement: S2 Text — Detailed mathematical descriptions of the statistical models used in the analysis. (PDF) [file pgph.0004704.s002.pdf]

S2 Text

Main effects of worries on mental health outcomes

For each study period (Period 1 and Period 2), we applied survey-weighted logistic regression models to examine the associations between pandemic-related worries and mental health outcomes in each country:

$$\operatorname{logit} P(Y = 1 \mid \boldsymbol{X}, \boldsymbol{Z}) = \beta_0 + \boldsymbol{\beta}_X \boldsymbol{X} + \boldsymbol{\beta}_Z \boldsymbol{Z}.$$

where Y represents self-reported frequent depression or anxiety, $\boldsymbol{X}$ is a vector of pandemic-related worries, and $\boldsymbol{Z}$ is a vector of covariates. In Period 1, $\boldsymbol{X}$ includes financial stress, food insecurity, and COVID-19-related illness concerns, while in Period 2, it includes only financial stress and food insecurity. The covariate set ($\boldsymbol{Z}$) consists of gender, age, education, rural–urban residential status, occupation, and calendar time.

Effect modification by vaccination

In Period 2, we fitted survey-weighted logistic regression models to assess whether COVID-19 vaccination modified the association between pandemic-related worries and mental health outcomes in each country:

$$\operatorname{logit} P(Y = 1 \mid \boldsymbol{X}, \boldsymbol{Z}, Vaccine) = \beta_0 + \boldsymbol{\beta}_X \boldsymbol{X} + \boldsymbol{\beta}_Z \boldsymbol{Z} + \beta_V Vaccine + \beta_{X_j V} X_j \times Vaccine.$$

Here, the definitions of Y, $\boldsymbol{X}$, and $\boldsymbol{Z}$ remain the same as in the main models. X_j represents one of the pandemic-related worries from vector $\boldsymbol{X}$, specifically either financial stress or food insecurity. Each model included an interaction term between only one worry variable (X_j) and vaccination status.

Effect modification by calendar time, gender, education, and rural-urban residential status

For each study period (Period 1 and Period 2), we fitted additional survey-weighted logistic regression models to investigate whether calendar time, gender, education, or rural–urban residential status modified the associations between pandemic-related worries and mental health outcomes in each country:

$$\operatorname{logit} P(Y = 1 \mid \boldsymbol{X}, \boldsymbol{Z}) = \beta_0 + \boldsymbol{\beta}_X \boldsymbol{X} + \boldsymbol{\beta}_Z \boldsymbol{Z} + \beta_{X_j Z_k} X_j \times Z_k.$$

Here, the definitions of Y, $\boldsymbol{X}$, $\boldsymbol{Z}$, and X_j remain the same as described previously. Z_k represents a single covariate (calendar time, gender, education, or rural–urban residential status) from the full covariate set $\boldsymbol{Z}$. Each model included the interaction term between only one worry variable (X_j) and one potential effect modifier (Z_k).
