# Supplementary material for: The role of financial stress, food insecurity, and COVID-19-related illness concerns shaping mental health in five South Asian countries during the pandemic (2020–2022): A secondary analysis of the online COVID-19 Trends and Impact Survey (CTIS) data
Source: PLOS Glob Public Health. 2025 Aug 8;5(8):e0004704. doi: 10.1371/journal.pgph.0004704 (PMC12334018; doi:10.1371/journal.pgph.0004704)
Supplement: S1 Table — Abbreviations: Period 1, June 27, 2020, to May 19, 2021; Period 2, May 20, 2021, to June 25, 2022. COVID-19-related health concerns were excluded from the surveys during Period 2. Note: a) The survey question on vaccination status was added on January 5, 2021. (PDF) [file pgph.0004704.s003.pdf]

S1 Table

S1 Table. Question phrasing and initial answers in the survey for Period 1 and Period 2.

		Period 1	Period 2
**Mental health**			
**Depression**	**Question**	During the last 7 days, how often did you feel so depressed that nothing could cheer you up?	
	**Responses**	1 = All the time<br>2 = Most of the time<br>3 = Some of the time<br>4 = A little of the time<br>5 = None of the time	
**Anxiety**	**Question**	During the past 7 days, how often did you feel so nervous that nothing could calm you down?	
	**Responses**	1 = All the time<br>2 = Most of the time<br>3 = Some of the time<br>4 = A little of the time<br>5 = None of the time	
**Worries about the pandemic**			
**Financial stress**	**Question**	How worried are you about your household's finances in the next month?	
	**Responses**	1 = Very worried<br>2 = Somewhat worried<br>3 = Not too worried<br>4 = Not worried at al	
**Food insecurity**	**Question**	How worried are you about having enough to eat in the next week?	
	**Responses**	1 = Very worried<br>2 = Somewhat worried<br>3 = Not too worried<br>4 = Not worried at al	
**COVID-19-related illness concerns**	**Question**	How worried are you that you or someone in your immediate family might become seriously ill from coronavirus (COVID-19)?	
	**Responses**	1 = Very worried<br>2 = Somewhat worried<br>3 = Not too worried<br>4 = Not worried at al	
**Demographics**			
**Gender**	**Question**	What is your gender?	
	**Responses**	1 = Male<br>2 = Female<br>3 = Other<br>4 = Prefer not to answer	
**Age**	**Question**	What is your age?	

S1 Table continued from previous page

	**Responses**	1 = 18-24 years<br>2 = 25-34 years<br>3 = 35-44 years<br>4 = 45-54 years<br>5 = 55-64 years<br>6 = 65-74 years<br>7 = 75 years or older	
**Education**	**Question**	How many years of education have you completed?	What is the highest level of education that you have completed?
	**Responses**	Open response: number validation	1 = No formal schooling<br>2 = Less than primary school<br>3 = Primary school completed<br>4 = Secondary school completed<br>5 = High school (or equivalent) completed<br>6 = College/ pre-university/ University completed<br>7 = University post-graduate degree completed
**Residential status**	**Question**	How many years of education have you completed?	
	**Responses**	1 = City<br>2 = Town<br>3 = Village or rural area	
**Occupation**	**Question**	What is the main activity of the business or organization in which you work?	
	**Responses**	1 = Agriculture<br>2 = Buying and selling<br>3 = Construction<br>4 = Education<br>5 = Electricity/water/gas/waste<br>6 = Financial/insurance/real estate services<br>7 = Health<br>8 = Manufacturing<br>9 = Mining<br>10 = Personal services<br>11 = Professional/scientific/technical activities<br>12 = Public administration<br>13 = Tourism<br>14 = Transportation<br>15 = Other	
**Vaccination status**[a]	**Question**	How you had a COVID-19 vaccination?	
	**Responses**	1 = Yes<br>2 = No<br>3 = I don't know	

Abbreviations: Period 1, June 27, 2020, to May 19, 2021; Period 2, May 20, 2021, to June 25, 2022. COVID-19-related health concerns were excluded from the surveys

during Period 2. Note: a) The survey question on vaccination status was added on January 5, 2021.
