# Supplementary material for: The role of financial stress, food insecurity, and COVID-19-related illness concerns shaping mental health in five South Asian countries during the pandemic (2020–2022): A secondary analysis of the online COVID-19 Trends and Impact Survey (CTIS) data
Source: PLOS Glob Public Health. 2025 Aug 8;5(8):e0004704. doi: 10.1371/journal.pgph.0004704 (PMC12334018; doi:10.1371/journal.pgph.0004704)
Supplement: S2 Table — Abbreviations: Period 2, May 20, 2021, to June 25, 2022. The results were obtained from separate unadjusted models that include vaccination status as the only covariate per model. All model types integrate survey weights within logistic regression. Only complete cases with no missing data on vaccination status, demographics, pandemic-related worries, and outcomes were included in the analysis. Odds ratios are displayed as estimates with corresponding 95% Wald confidence intervals in the form of estimates [95% confidence interval], using a robust sandwich estimator for variance calculation. Significant odds ratios from Wald tests (significance level: 0.05) are highlighted in bold. (PDF) [file pgph.0004704.s004.pdf]

S2 Table

S2 Table. Effects of vaccination status on mental health and pandemic-related worries across five South Asian countries during Period 2 (N = 234,149), post-weighting.

	Vaccination status				
	**Bangledash (N = 33,914)**	**India (N = 151,804)**	**Nepal (N = 12,054)**	**Pakistan (N = 28,915)**	**Sri Lanka (N = 7,462)**
**Depression**	**0.60 [0.53, 0.68]**	**0.67 [0.62, 0.74]**	**0.56 [0.44, 0.71]**	**0.65 [0.55, 0.77]**	**0.62 [0.48, 0.80]**
**Nervousness**	**0.69 [0.62, 0.77]**	**0.56 [0.43, 0.72]**	**0.61 [0.51, 0.73]**	0.79 [0.61, 1.02]	**0.62 [0.53, 0.72]**
**Financial stress**	**0.50 [0.45, 0.56]**	**0.49 [0.46, 0.52]**	**0.43 [0.36, 0.51]**	**0.65 [0.57, 0.74]**	**0.61 [0.49, 0.75]**
**Food insecurity**	**0.41 [0.34, 0.50]**	**0.54 [0.50, 0.58]**	**0.55 [0.41, 0.74]**	**0.63 [0.53, 0.74]**	**0.52 [0.37, 0.71]**

Abbreviations: Period 2, May 20, 2021, to June 25, 2022. The results were obtained from separate unadjusted models that include vaccination status as the only covariate per model. All model types integrate survey weights within logistic regression. Only complete cases with no missing data on vaccination status, demographics, pandemic-related worries, and outcomes were included in the analysis. Odds ratios are displayed as estimates with corresponding 95% Wald confidence intervals in the form of estimates [95% confidence interval], using a robust sandwich estimator for variance calculation. Significant odds ratios from Wald tests (significance level: 0.05) are highlighted in bold.
