# Supplementary material for: The role of financial stress, food insecurity, and COVID-19-related illness concerns shaping mental health in five South Asian countries during the pandemic (2020–2022): A secondary analysis of the online COVID-19 Trends and Impact Survey (CTIS) data
Source: PLOS Glob Public Health. 2025 Aug 8;5(8):e0004704. doi: 10.1371/journal.pgph.0004704 (PMC12334018; doi:10.1371/journal.pgph.0004704)

S1 Fig

S1 Fig. Overview of data processing. This secondary analysis draws on data from the COVID-19 Trends and Impact Survey, a global online survey administered to adult Facebook active users. The study period spans from June 27, 2020, to June 25, 2022, with a major survey revision on May 20, 2021, marking two distinct analytical phases. Descriptive analyses include the full sample from five South Asian countries (Bangladesh, India, Nepal, Pakistan, and Sri Lanka), while statistical analyses are limited to respondents with complete data.

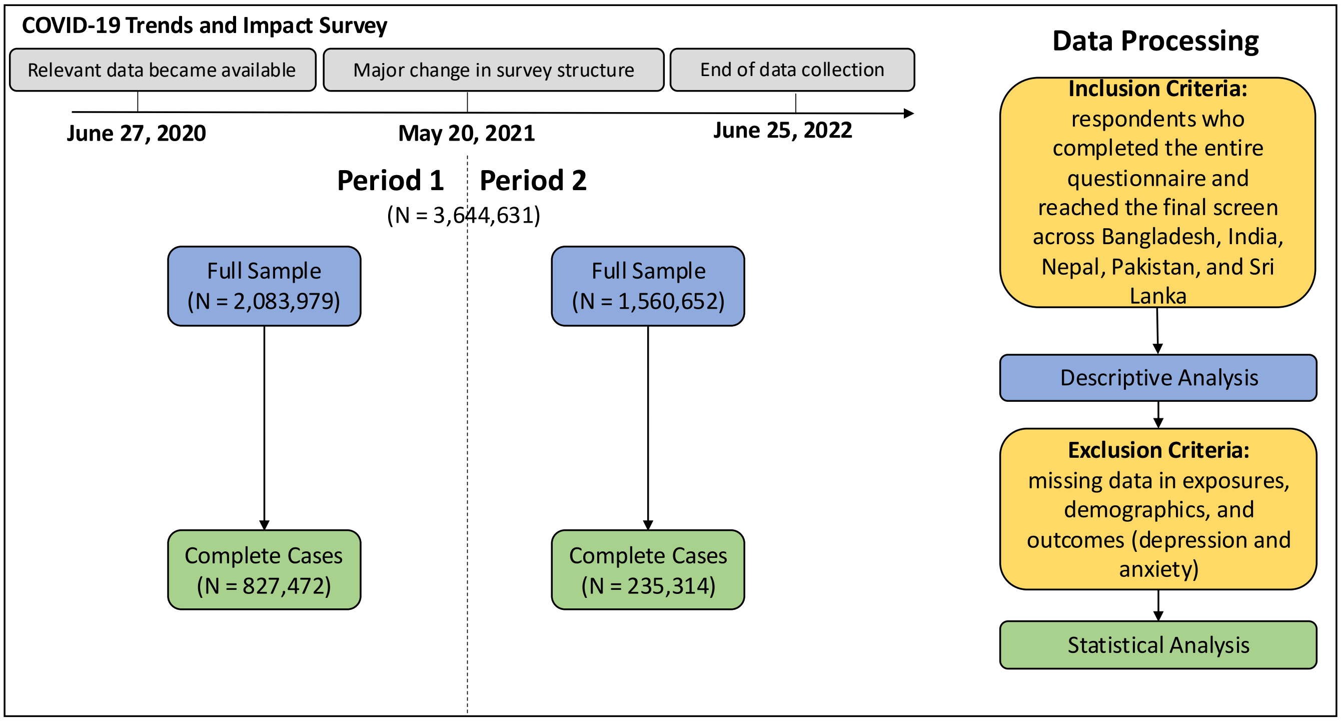

Supplement: S1 Fig — This secondary analysis draws on data from the COVID-19 Trends and Impact Survey, a global online survey administered to adult Facebook active users. The study period spans from June 27, 2020, to June 25, 2022, with a major survey revision on May 20, 2021, marking two distinct analytical phases. Descriptive analyses include the full sample from five South Asian countries (Bangladesh, India, Nepal, Pakistan, and Sri Lanka), while statistical analyses are limited to respondents with complete data. (PDF) [file pgph.0004704.s005.pdf]
