# Supplementary material for: The role of financial stress, food insecurity, and COVID-19-related illness concerns shaping mental health in five South Asian countries during the pandemic (2020–2022): A secondary analysis of the online COVID-19 Trends and Impact Survey (CTIS) data
Source: PLOS Glob Public Health. 2025 Aug 8;5(8):e0004704. doi: 10.1371/journal.pgph.0004704 (PMC12334018; doi:10.1371/journal.pgph.0004704)

S4 Fig

S4 Fig. Effects of pandemic-related worries on mental health between gender across five South Asian countries in Period 1 (N = 827,472) and Period 2 (N = 235,314), post-weighting. Abbreviations: Period 1, June 27, 2020, to May 19, 2021; Period 2, May 20, 2021, to June 25, 2022; OR, odds ratio; CI, confidence interval. COVID-19-related health concerns were excluded from the surveys during Period 2. Separate weighted logistic regression models were fitted for complete cases from each period, including pandemic-related worries (financial stress, food insecurity, and COVID-19-related illness concerns), demographics (gender, age, education, rural-urban residential status, and occupation), and calendar time (categorized by month and year) as covariates. Interaction terms between gender and one worry variable were included in each model. The results present odds ratios with their corresponding 95% Wald confidence intervals. Robust sandwich estimators were applied for variance estimation. The x-axis is presented on a logarithmic scale to improve visualization. The vertical black lines at an odds ratio of 1 represent the null.

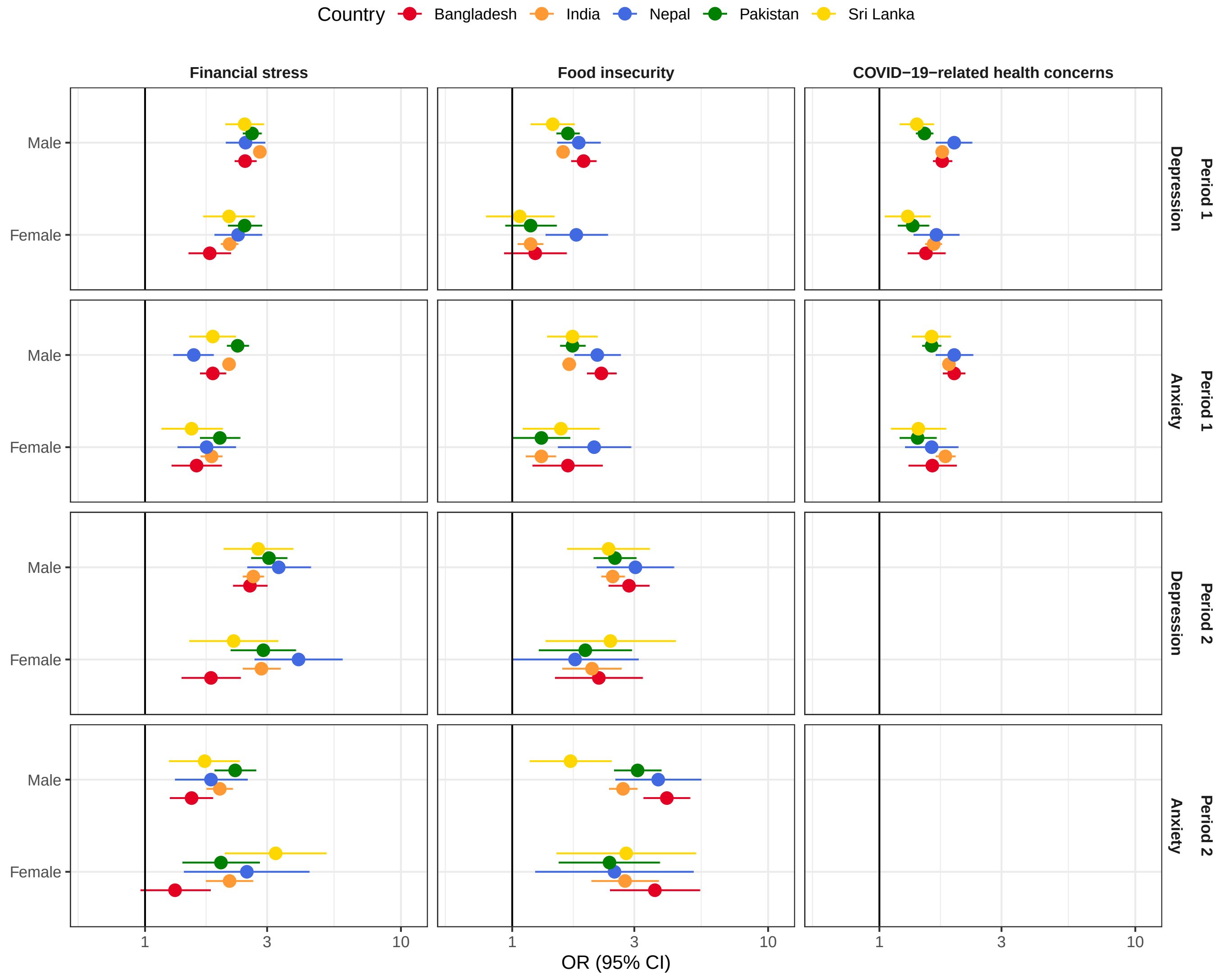

Supplement: S4 Fig — Abbreviations: Period 1, June 27, 2020, to May 19, 2021; Period 2, May 20, 2021, to June 25, 2022; OR, odds ratio; CI, confidence interval. COVID-19-related health concerns were excluded from the surveys during Period 2. Separate weighted logistic regression models were fitted for complete cases from each period, including pandemic-related worries (financial stress, food insecurity, and COVID-19-related illness concerns), demographics (gender, age, education, rural-urban residential status, and occupation), and calendar time (categorized by month and year) as covariates. Interaction terms between gender and one worry variable were included in each model. The results present odds ratios with their corresponding 95% Wald confidence intervals. Robust sandwich estimators were applied for variance estimation. The x-axis is presented on a logarithmic scale to improve visualization. The vertical black lines at an odds ratio of 1 represent the null. (PDF) [file pgph.0004704.s008.pdf]
