# Supplementary material for: The role of financial stress, food insecurity, and COVID-19-related illness concerns shaping mental health in five South Asian countries during the pandemic (2020–2022): A secondary analysis of the online COVID-19 Trends and Impact Survey (CTIS) data
Source: PLOS Glob Public Health. 2025 Aug 8;5(8):e0004704. doi: 10.1371/journal.pgph.0004704 (PMC12334018; doi:10.1371/journal.pgph.0004704)

S6 Fig

S6 Fig. Effects of pandemic-related worries on mental health between rural-urban residential statuses across five South Asian countries in Period 1 (N = 827,472) and Period 2 (N = 235,314), post-weighting. Abbreviations: Period 1, June 27, 2020, to May 19, 2021; Period 2, May 20, 2021, to June 25, 2022; OR, odds ratio; CI, confidence interval. COVID-19-related health concerns were excluded from the surveys during Period 2. Separate weighted logistic regression models were fitted for complete cases from each period, including pandemic-related worries (financial stress, food insecurity, and COVID-19-related illness concerns), demographics (gender, age, education, rural-urban residential status, and occupation), and calendar time (categorized by month and year) as covariates. Interaction terms between rural-urban residential statuses and one worry variable were included in each model. The results present odds ratios with their corresponding 95% Wald confidence intervals. Robust sandwich estimators were applied for variance estimation. The x-axis is presented on a logarithmic scale to improve visualization. The vertical black lines at an odds ratio of 1 represent the null.

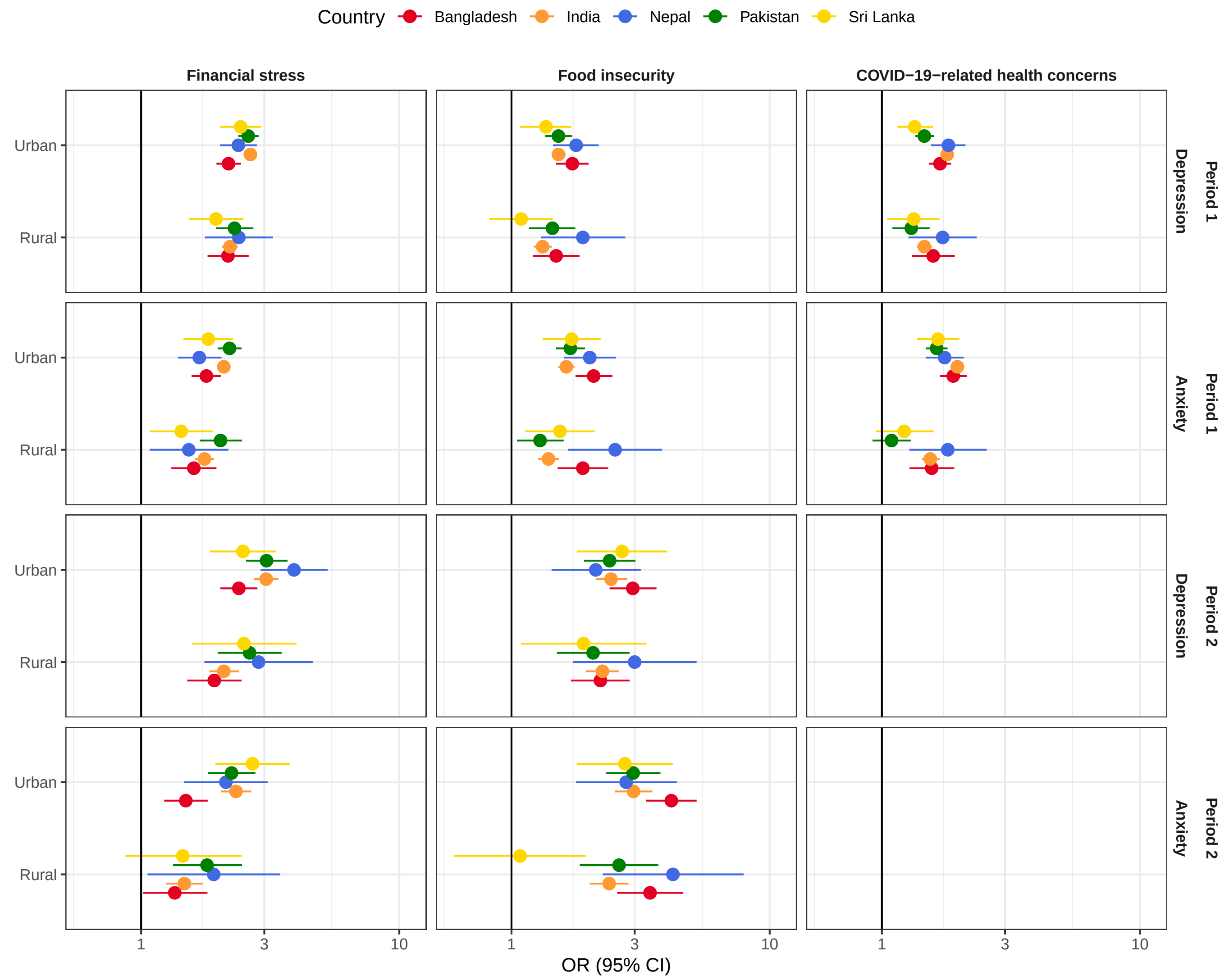

Supplement: S6 Fig — Abbreviations: Period 1, June 27, 2020, to May 19, 2021; Period 2, May 20, 2021, to June 25, 2022; OR, odds ratio; CI, confidence interval. COVID-19-related health concerns were excluded from the surveys during Period 2. Separate weighted logistic regression models were fitted for complete cases from each period, including pandemic-related worries (financial stress, food insecurity, and COVID-19-related illness concerns), demographics (gender, age, education, rural-urban residential status, and occupation), and calendar time (categorized by month and year) as covariates. Interaction terms between rural-urban residential statuses and one worry variable were included in each model. The results present odds ratios with their corresponding 95% Wald confidence intervals. Robust sandwich estimators were applied for variance estimation. The x-axis is presented on a logarithmic scale to improve visualization. The vertical black lines at an odds ratio of 1 represent the null. (PDF) [file pgph.0004704.s010.pdf]
