# Supplementary material for: The role of financial stress, food insecurity, and COVID-19-related illness concerns shaping mental health in five South Asian countries during the pandemic (2020–2022): A secondary analysis of the online COVID-19 Trends and Impact Survey (CTIS) data
Source: PLOS Glob Public Health. 2025 Aug 8;5(8):e0004704. doi: 10.1371/journal.pgph.0004704 (PMC12334018; doi:10.1371/journal.pgph.0004704)
Supplement: Attachment — Submitted filename: Response to Reviewers.pdf [file pgph.0004704.s012.pdf]

Response to Reviewers

Title: The role of financial stress, food insecurity, and COVID-19-related illness concerns shaping mental health in five South Asian countries during the pandemic (2020–2022): A secondary analysis of the online COVID-19 Trends and Impact Survey (CTIS) data

Authors: Youqi Yang, Lauren Zimmermann, Santanu Pramanik, Brian Wahl, and Bhramar Mukherjee

Manuscript ID: PGPH-D-25-00676

Reviewer 1

General comments	Kindly check the language, punctuation, and remove any redundancy.
Abstract	The abstract is well-written.
Introduction	The introduction is well-written.
Materials and Methods	The survey sample is not clearly defined. The manuscript should specify how the sample size was determined and provide a clear explanation of the sampling strategy. Additionally, the data collection process for the reported sample size (N = 3,644,631) needs to be described in detail, including the sources, methods used, and any inclusion or exclusion criteria applied.
Results	The Results section is clearly written and effectively presents the key findings.
Discussion	The discussion is well-written.
Conclusion	The conclusion is well-written.
References	The references appear to be well-organized.

Response: Thank you for your comments. This study is a secondary analysis of data from the global COVID-19 Trends and Impact Survey (CTIS), administered by Meta/Facebook in collaboration with the University of Maryland and Carnegie Mellon University (Astley et al., 2021). The survey was distributed daily to a random sample of active Facebook users across more than 130 countries and regions. Our analysis covers the period from June 27, 2020—when data relevant to our research question first became available—through June 25, 2022, when CTIS data collection concluded. The inclusion criterion was that samples were collected from five South Asian countries: Bangladesh, India, Nepal, Pakistan, and Sri Lanka. For the statistical analyses, we included respondents with complete data on depression, anxiety, pandemic-related worries, and key demographic variables (gender, age, education, rural-urban residential status,

occupation, and vaccination status). Details about the survey data and sampling procedures are provided in the "Data Source" section (Lines 82–114).

We have included the following figure as supplementary S1 Fig in the appendix to visualize the data processing and curation steps that led us to the analytic sample.

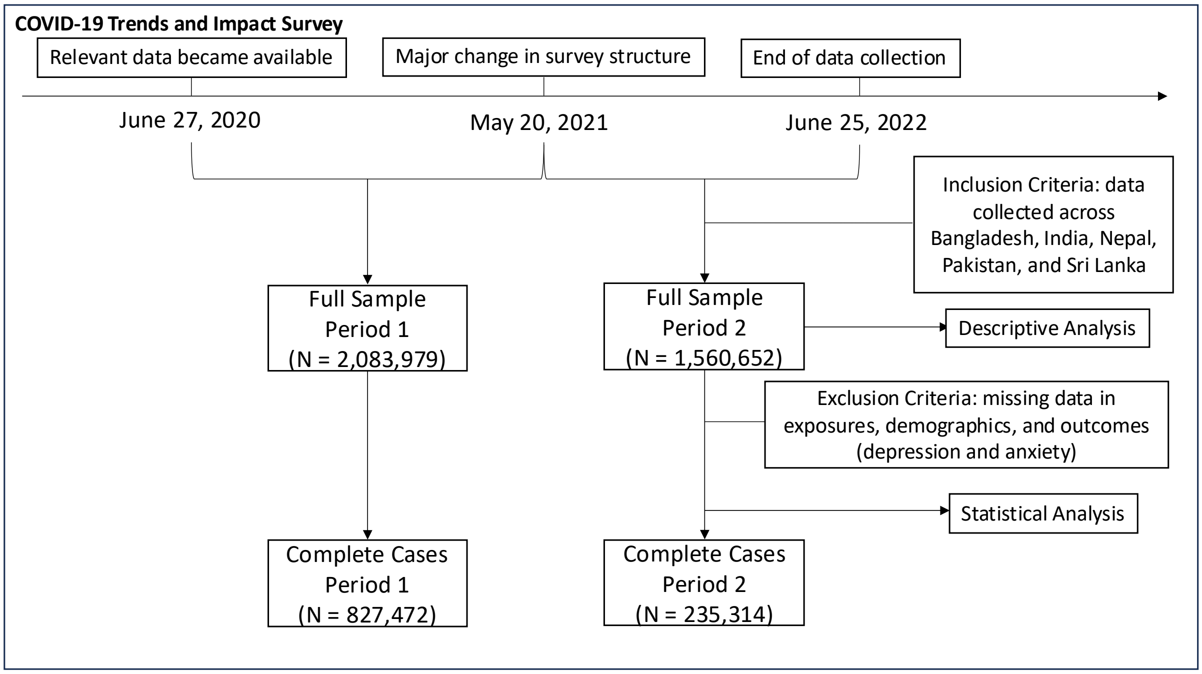

"**S1 Fig. Overview of data processing.** This secondary analysis draws on data from the COVID-19 Trends and Impact Survey, a global online survey administered to adult Facebook active users. The study period spans from June 27, 2020, to June 25, 2022, with a major survey revision on May 20, 2021, marking two distinct analytical phases. Descriptive analyses include the full sample from five South Asian countries (Bangladesh, India, Nepal, Pakistan, and Sri Lanka), while statistical analyses are limited to respondents with complete data."

Reviewer 2

Dear Author,

Thank you for your work and for submitting this manuscript. I have a few comments that I hope will help strengthen your paper:

1. Could the title more clearly reflect the type of study conducted? I had to read into the manuscript to determine the study design. Including this information in the title could improve clarity and accessibility for readers.

Response: Thank you for your helpful suggestion. We have revised the title to more clearly reflect the study design, specifying that this is a secondary analysis of online survey data from Facebook/Meta platform. The new title is:

The role of financial stress, food insecurity, and COVID-19-related illness concerns shaping mental health in five South Asian countries during the pandemic (2020–2022): A secondary analysis of the online COVID-19 Trends and Impact Survey (CTIS) data

2. Lines 36–40 Pandemic-Related Worries: The connection between financial stress, food insecurity, and the pandemic is not well established in the current manuscript. These issues certainly exist independently of the pandemic. If the intent is to highlight that these concerns worsened or became more prominent due to the pandemic, please provide supporting evidence or references to substantiate this claim.

Response: Thank you for your comment. We have added relevant literature to contextualize the relationship between worry and the pandemic. For example, a cross-sectional survey in Bangladesh found that 90% of households experienced some degree of food insecurity during the pandemic (Das et al., 2020). Similarly, a longitudinal study in India reported that 62% of previously food-secure households became food insecure in 2020 (Kesar et al., 2021). We have also incorporated findings from the 2021 report by the Food and Agriculture Organization of the United Nations to further support these observations (Food and Agriculture Organization of the United Nations, 2021).

We have added to the “Introduction” section, on lines 26-34, the following:

“In response to the COVID-19 pandemic, all five South Asian countries implemented nationwide or regionally enforced lockdowns during 2020 and 2021 (27). These disruptions to routine economic activities substantially constrained household resources, exacerbating financial hardship and contributing to increased food insecurity across the region (32). In Bangladesh, a cross-sectional survey found that 90% of households experienced some level of food insecurity during the pandemic (33). Similarly, a longitudinal study in India reported that 62% of previously food-secure households became food insecure in 2020 (34). Comparable patterns of financial hardship and declining food security were also observed in other countries (35).”

3. Line 46 Online Survey and Response Bias: You mention the use of an online survey, yet this does not appear in your limitations section. Given that the study

population includes a substantial proportion of rural and potentially lower-educated individuals, this raises concerns about response bias and representativeness. How was this addressed or mitigated? If you have data or references indicating that mobile phone and internet usage in the surveyed areas is sufficient to support representative online data collection, please include this to justify your methodology.

Response: Thank you for your thoughtful comment. There are two primary limitations associated with using an online survey. First, selection bias may arise due to the self-selecting nature of participation. While Facebook implemented a two-stage weighting procedure to partially address this concern, the demographic composition of respondents—particularly for variables not included in the weighting process, such as education level and rural-urban residential status—remains meaningfully different from national census benchmarks. Second, the outcomes are self-reported, which limits the ability to interpret the findings as clinical or medical assessments.

We have added to the “Limitation” section of the “Discussion” section, on lines 468-478, the following to expand on the limitations as noted above:

“This study has several limitations. First, the use of a non-probability sample distributed via a social media platform introduces inherent selection bias due to participants' self-selection (53). Despite the weighting adjustments provided by Facebook, this bias could not be fully corrected, particularly for variables not included in the weighting process. For example, the sample consistently overrepresented individuals with a high school education or higher, as well as those living in urban areas across all five countries, diverging from national census benchmarks. This limitation, observed in prior studies as well (54,75), may restrict the generalizability of our findings to the broader adult population. Second, both depression and anxiety were assessed using self-reported symptom frequencies, which do not equate to clinical diagnoses. Demographic variables were also self-reported, introducing the possibility of reporting bias, measurement error, and misclassification.”

4. Figure 3 Contradictory Findings: Figure 3 suggests higher odds ratios for outcome variables among vaccinated and urban individuals, which contrasts with the narrative in lines 434–438. This discrepancy is not clearly addressed in the text. Could you elaborate on this or provide a potential explanation?

Response: Thank you for your comment. On Lines 434–438, we report the main effect of vaccination where it is associated with reductions in both self-reported anxiety and depression (our outcome variable). It is also associated with pandemic related worries (our exposure variable). Figure 3, by contrast, examines vaccination as an effect modifier: here, vaccinated individuals show a stronger association between

worries and self-reported mental health problems. One plausible interpretation is that people who get vaccinated, do so because of their worries and there is a possible reverse causality. An alternative interpretation is that vaccination was perceived by this vaccinated group as “the blanket solution” for returning to normalcy—so when disruptions persisted (e.g., lockdowns, breakthrough cases, economic hardship), vaccinated people experienced a greater gap between expectations and reality, which may have amplified the psychological impact of their worries.

To further confirm the role of vaccination as an effect modifier, we fit two separate models for vaccinated and unvaccinated individuals in Period 2 and examined the associations between worry variables and mental health within each group. The results, shown below, are largely consistent with those presented in Figure 3.

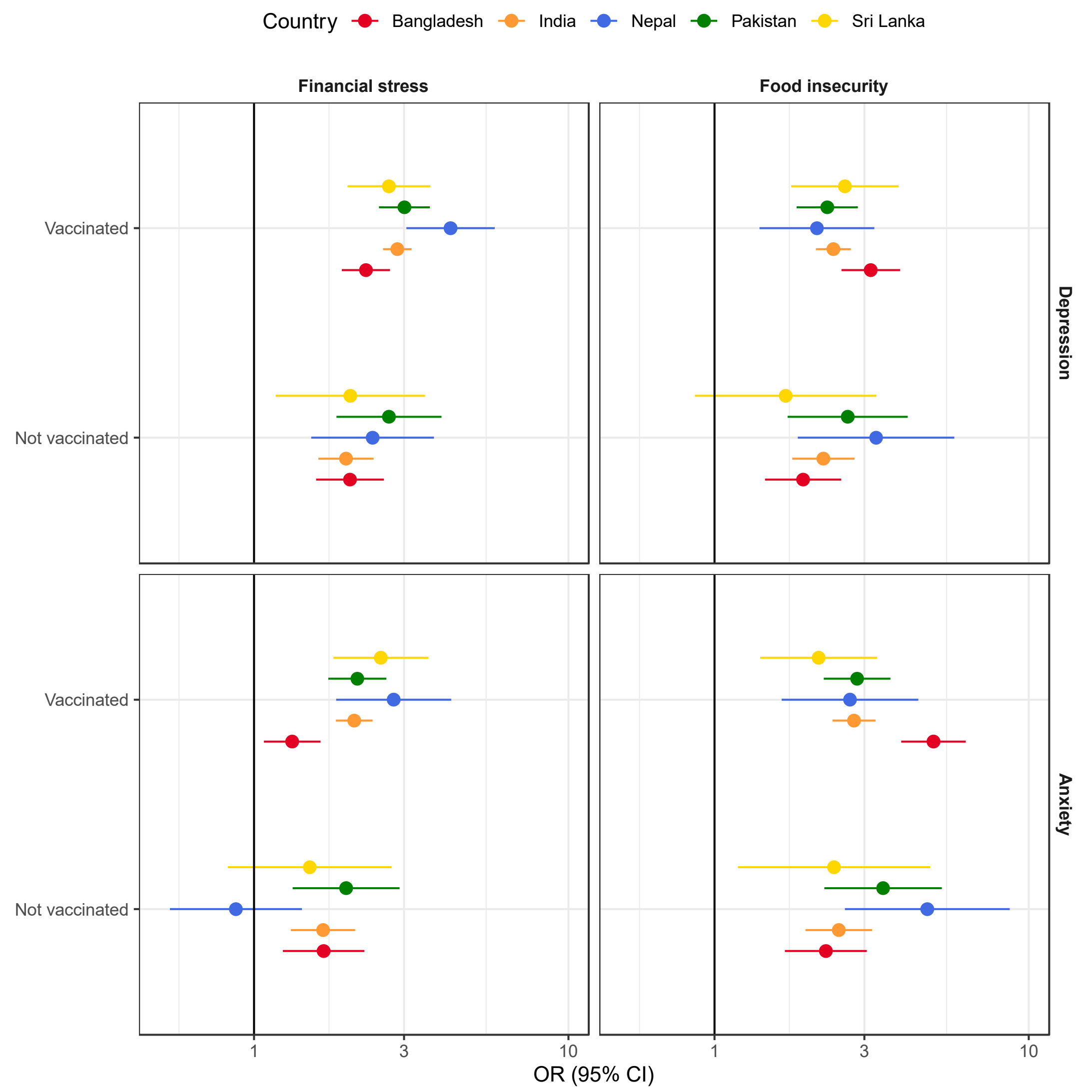

Effects of pandemic-related worries on mental health stratified by vaccination statuses across five

South Asian countries during Period 2 (N = 234,149), post-weighting.

In India, pandemic-related worries were more strongly associated with self-reported frequent depression and anxiety among urban residents compared to their rural counterparts; however, this pattern was not observed in the other four countries (see S6 Fig). Our previous work in India found that living in rural areas was linked to better mental health outcomes (Yang et al., 2024). The heightened vulnerability among urban populations may reflect greater exposure to social isolation policies, particularly among migrant workers.

We have added the "Vaccination status" section within the "Effect modification by time and participant characteristics" section of the Discussion on lines 442-458 as follows:

"An intriguing result of our study is that, in four of the five South Asian countries examined, vaccination status was associated with improved mental health outcomes—mirroring U.S. findings of reduced distress (71,72) and lower food insecurity (73) (see S2 Table). However, vaccination status modified the relationship between pandemic-related worries and adverse mental health outcomes. In other words, while vaccinated individuals generally reported lower levels of pandemic-related worries, depression, and anxiety, the strength of association between their worry and mental health was larger than the corresponding association among unvaccinated individuals. One possible explanation is that individuals who choose to get vaccinated do so because of heightened worries and concerns, raising the potential for reverse causality. It could also be that vaccination was widely viewed as "the solution" for restoring normal life; when pandemic disruptions persisted, vaccinated people may have experienced a more pronounced gap between expectations and reality. To our knowledge, this dual role of vaccination—as a protective variable for depression/anxiety when treated as a main effect but an effect modifier of the relationship between depression/anxiety and the set of worries is a new finding that needs further understanding and replication."

Thank you again for your contributions, and I look forward to seeing your revised manuscript.

Unique Citations in the Response to the Reviewers

Astley CM, Tuli G, Mc Cord KA, Cohn EL, Rader B, Varrelman TJ, et al. Global monitoring of the impact of the COVID-19 pandemic through online surveys sampled from the Facebook user base. Proceedings of the National Academy of Sciences. 2021;118(51):e2111455118.

Das S, Rasul MG, Hossain MS, Khan AR, Alam MA, Ahmed T, et al. Acute food insecurity and short-term coping strategies of urban and rural households of Bangladesh during the lockdown period of COVID-19 pandemic of 2020: report of a cross-sectional survey. BMJ open. 2020;10(12):e043365.

Food and Agriculture Organization of the United Nations. The State of Food Security and Nutrition in the World 2021: Transforming food systems for food security, improved nutrition and affordable healthy diets for all; 2021. https://www.fao.org/interactive/state-of-food-security-nutrition/2021/en/.

Kesar S, Abraham R, Lahoti R, Nath P, Basole A. Pandemic, informality, and vulnerability: Impact of COVID-19 on livelihoods in India. Canadian Journal of Development Studies/Revue canadienne d'´etudes du d´eveloppement. 2021;42(1-2):145–164.

Yang Y, Sun A, Zimmermann L, Mukherjee B. Impact of pandemic-related worries on mental health in India from 2020 to 2022. npj Mental Health Research. 2024;3(1):57.
