# Supplementary material for: The role of financial stress, food insecurity, and COVID-19-related illness concerns shaping mental health in five South Asian countries during the pandemic (2020–2022): A secondary analysis of the online COVID-19 Trends and Impact Survey (CTIS) data
Source: PLOS Glob Public Health. 2025 Aug 8;5(8):e0004704. doi: 10.1371/journal.pgph.0004704 (PMC12334018; doi:10.1371/journal.pgph.0004704)
Supplement: Attachment — Submitted filename: Response to reviewers_PGH.pdf [file pgph.0004704.s013.pdf]

Response to Reviewers

Title: The role of financial stress, food insecurity, and COVID-19-related illness concerns shaping mental health in five South Asian countries during the pandemic (2020–2022): A secondary analysis of the online COVID-19 Trends and Impact Survey (CTIS) data

Authors: Youqi Yang, Lauren Zimmermann, Santanu Pramanik, Brian Wahl, and Bhramar Mukherjee

Manuscript ID: PGPH-D-25-00676

Reviewer 1

The topic is interesting and well appreciated. Kindly revise the manuscript before it is considered for publication.

Abstract:
This section is well written.

Introduction:
This section still requires improvement. Please ensure a logical flow based on the study objectives. Develop the section by incorporating relevant previous studies as part of the literature review. Clearly state the problem and rationale, explaining why this study is timely and necessary.

Response: Thank you for your comment. Based on your feedback, we have restructured the introduction to describe: (a) the broader context of studying the relationship between day to day worries and mental health; (b) the need to study this relationship during the pandemic; (c) the paucity of literature both during normal times and pandemic times in South Asia; (d) the contribution of the current study in addressing the existing knowledge gap and towards defining future pandemic policies, balancing between lives and livelihoods. We hope you find this reorganization more logically coherent and fluent.

We have reorganized the “Introduction” section, on Lines 2-74, to reflect this change (quoted below):

“Depression and anxiety are among the most widespread mental health conditions globally [1]. Many researchers have investigated the subjective worries—such as financial stress, food insecurity, and health-related concerns—that are commonly associated with

these conditions. A recent review of 40 observational studies found a consistent positive association between financial stress and depression [2]. In parallel, a global cross-sectional study using data from 149 countries reported that food insecurity was linked to poorer mental health [3]. During a public health crisis, such as widespread infectious disease outbreaks, health-related illness concerns have been reported to be associated with elevated rates of psychological stress and disorders; this pattern was evident during the 2013–2016 Ebola outbreak in West Africa [4]. Examining potential social and economic effect modifiers of these associations can provide deeper insight into subgroup-specific vulnerabilities, as subjective experiences of worry often vary across individuals with different backgrounds, even when objective circumstances are similar. For instance, a study among U.S. college students found that the association between financial stress and symptoms of generalized anxiety was stronger among women than among men [5].

The dramatic spread of the COVID-19 pandemic from 2020-2022 profoundly affected not only physical but also mental well-being worldwide [6]. Researchers have consistently documented a rise in depression and anxiety among the general public during the pandemic [7-13]. A systematic review of 5,683 data sources estimated that the pandemic contributed to an additional 53.2 (95% confidence intervals, CI: [44.8, 62.9]) million cases of major depressive disorders and 76.2 (95% CI: [64.3, 90.6]) million cases of anxiety disorders worldwide [14]. Alongside the health crisis, the pandemic also severely disrupted economic systems and food supply chains. In response, a growing number of studies have explored how worries are associated with mental health outcomes in the context of COVID-19. Global evidence indicates that financial stress [15-20], food insecurity [21-27], and COVID-19-related health concerns [20,25,28,29] all played significant roles in the deterioration of mental health during this period. However, the data and evidence are not equitably distributed across all regions in the world.

This study focuses on five low- and middle-income countries (LMICs) from South Asia—Bangladesh, India, Nepal, Pakistan, and Sri Lanka—which together account for 23.5% of the global population. Systematic reviews have indicated increased burden of poor mental health outcomes among the general population in South Asia during the COVID-19 pandemic [30,31]. In response to the health crisis, all five countries implemented nationwide or regionally enforced strict public health interventions including lockdowns during 2020 and 2021 [32]. These disruptions to routine economic activities substantially constrained household resources, exacerbating poverty and contributing to increased food insecurity across the region [33-36]. Despite these documented impacts, there is limited quantitative research examining how pandemic-related worries are linked to mental health in the general adult population using comparable measurements and metrics across these countries over time. Most of the existing studies have focused on specific subpopulations within individual countries, such as rural women [37] and daily

wage earners [38] in Bangladesh, small business entrepreneurs [39] and college students [40] in India, and urban informal sector laborers [41] in Pakistan. Most of these studies are cross-sectional and do not have repeated measures over the course of the pandemic. Furthermore, the literature on potential effect modifiers of the associations between worries and mental health remains very sparse. Where such analyses do exist, they have largely been conducted in high-income countries (HICs), including Canada [42], Portugal [43], and the United States [5,44], leaving a critical gap in understanding these dynamic interplays in South Asia and LMICs more generally.

Our study is a secondary analysis of the COVID-19 Trends and Impact Survey (CTIS), a large-scale global dataset covering a wide range of COVID-19-related topics from the onset through the later stages of the pandemic (2020–2022). CTIS has been widely recognized as a valuable resource for examining mental health outcomes [45–47] and their associations with pandemic-related worries [48, 49], particularly in HICs. Researchers have leveraged this dataset both to study specific subpopulations—such as teachers [45]—and to analyze trends in the general adult population [47]. The availability of individual-level data, collected over time and across regions, makes CTIS especially valuable, and such granular and comparable data remain relatively rare in South Asia.

This study builds on our prior work in India [50] by expanding the scope to a regional analysis of five South Asian countries. We examine the associations between pandemic-related worries and depression/anxiety among the general population during the period from 2020 to 2022. By analyzing data across Bangladesh, India, Nepal, Pakistan, and Sri Lanka, we aim to uncover both commonalities and differences in these associations across countries that are geographically proximate yet diverse in terms of demographic profiles, socioeconomic conditions, healthcare systems, and pandemic responses [32, 51-53]. In addition, we investigate how key socioeconomic factors—including vaccination status, self-reported gender, education, and rural-urban residential status—modify the relationship between worries and mental health. To our knowledge, this is the first cross-country analysis in LMIC settings to examine such effect modification, offering novel insights into the heterogeneity of mental health risks."

The following lines should be relocated to the Materials and Methods section:

"Our study used data from the COVID-19 Trends and Impact Survey (CTIS), which was conducted on the Facebook social media platform in collaboration with Meta/Facebook, Carnegie Mellon University (for the United States) [45], and the University of Maryland (globally) [46]. This survey, with its broad coverage of COVID-19-related topics, has proven to be a valuable resource for understanding mental health [47–49] and its associations with pandemic-related worries [50, 51]."

Response: Thank you for your comment. We have relocated this part to the "Materials and Methods" section, on Lines 87-90, the following.

"We used individual-level data from CTIS, a large-scale, daily repeated cross-sectional survey. The survey was conducted by Meta/Facebook in collaboration with Carnegie Mellon University for the United States [54], and the University of Maryland for all other participating countries [55]."

Materials and Methods:
Please elaborate on how the number of respondents (N = 3,644,631) was determined and how they were distributed across the five countries. What were the inclusion criteria for respondents?

Response: Thank you for your comment. Our inclusion criterion required that participants were from one of five South Asian countries—Bangladesh, India, Nepal, Pakistan, or Sri Lanka—and completed the global CTIS between June 27, 2020 (when the questions regarding pandemic-related worries became available), and June 25, 2022 (the end of data collection). We analyzed the full sample provided by Facebook, defined as respondents who completed the entire questionnaire and received the final "Thank you" message. The sample size for each country by period is shown below and is also available in Table 1 and Table 2 of the manuscript.

Country	Sample size	Proportion
Bangladesh	399,499	11%
India	2,576,174	71%
Nepal	200,152	5%
Pakistan	380,008	10%
Sri Lanka	88,798	2%
Total	3,644,631	100%

We have added to "Materials and Methods" section, on Lines 107-111, the following:

"Our analysis spanned from June 27, 2020, the date when questions about pandemic-related worries were first introduced into the survey, to June 25, 2022, the final day of the survey. Our full sample included respondents who completed the entire questionnaire and reached the final screen (N = 3,644,631 across five South Asian countries)."

We have updated S1 Fig in the appendix for visualizations of the data procession steps as presented below.

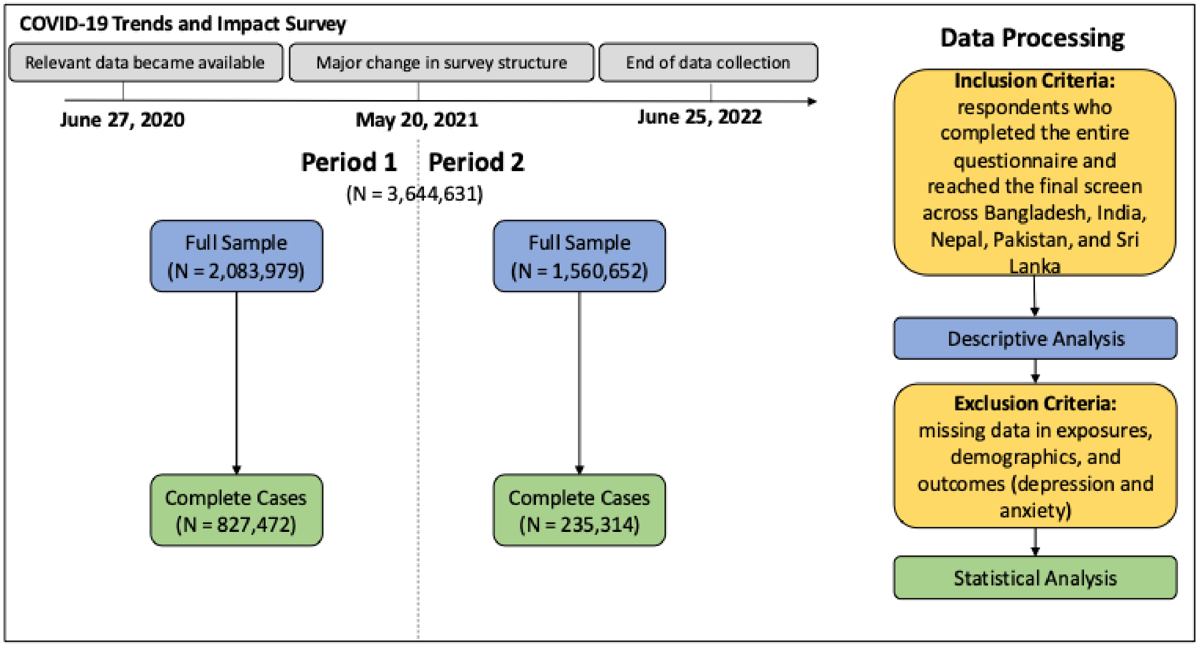

"**S1 Fig. Overview of data processing.** This secondary analysis draws on data from the COVID-19 Trends and Impact Survey, a global online survey administered to adult Facebook active users. The study period spans from June 27, 2020, to June 25, 2022, with a major survey revision on May 20, 2021, marking two distinct analytical phases. Descriptive analyses include the full sample from five South Asian countries (Bangladesh, India, Nepal, Pakistan, and Sri Lanka), while statistical analyses are limited to respondents with complete data."

Also, describe in detail the process of using the Facebook platform for data collection.

Response: Thank you for your comment. The sampling frame consists of Facebook's monthly active adult users. Each day, Facebook/Meta randomly invites a subset of these users to participate in the CTIS via a banner displayed at the top of their News Feed. Interested users are redirected off the Facebook platform to complete the survey. The survey itself is administered through the web-based Qualtrics platform, hosted by the Delphi Group at Carnegie Mellon University (CMU) for U.S. respondents, and by the Social Data Science Center at the University of Maryland (UMD) for participants in all other countries. As data users, we receive access to de-identified microdata only. A more detailed description of the global (non-U.S.) CTIS is provided in Astley et al. (2021). Below is a figure cited from that paper to illustrate the collection process.

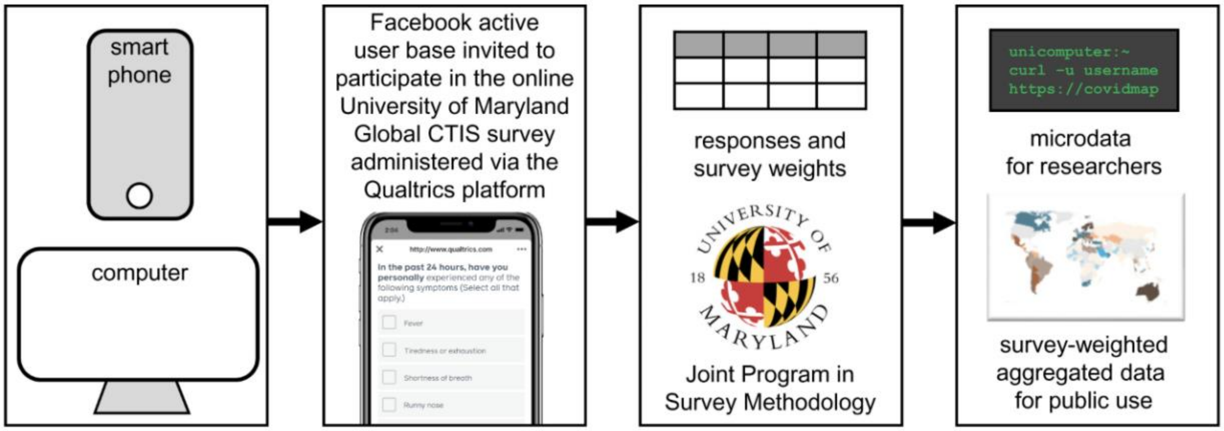

Source: Astley et al. (2021)

A brief description of the CTIS survey methodology is available in the "Materials and Methods" section.

Results:
This section is well written.

Discussion:
This section needs further improvement. Please strengthen it by justifying your findings with relevant and recent studies.

Response: We appreciate your suggestion to contextualize our findings within the broader literature. In response, we have restructured the Discussion section to explicitly connect our results to relevant prior work. The revised section now highlights three distinct contributions: (a) how our findings relate to existing research on mental health outside the pandemic context; (b) how they align with global studies conducted during the COVID-19 pandemic; and (c) how they extend the limited body of pandemic-related mental health research from South Asia. We have also highlighted the leverage of CTIS as a data source, using homogeneous and consistent data collection methods and measurement instruments across the five countries.

To support this revised structure, we have added several citations to underscore the relevance of our findings. First, we incorporated literature examining socioeconomic factors as potential effect modifiers in the associations between worries and mental health in non-pandemic settings (Tran et al., 2018; Hamilton et al., 2019; Viseu et al., 2021; Ryu et al., 2023). Second, we cited studies from outside South Asia that document similar associations between pandemic-related worries and mental health outcomes (Wilson et al., 2020; Fang et al., 2021; Wright et al., 2021). To the best of our knowledge, there is

currently no literature examining potential effect modifiers in these associations within the context of the COVID-19 pandemic.

We have reorganized the “Discussion” section, on Lines 406-478 in the following way:

How do our findings relate to the literature during non-pandemic times?

Our primary finding indicates that all three worries—financial stress, food insecurity, and COVID-19-related illness concerns—were significantly associated with increased levels of depression and anxiety in all five countries, after adjusting for demographics and calendar time. This pattern aligns with the pre-pandemic research linking economic and health-related stressors to poor mental health outcomes [2-4].

Importantly, our study is among the first to examine the role of socioeconomic factors as potential effect modifiers of these associations in LMIC settings. We found notable heterogeneity in how worries influenced mental health across different socioeconomic subgroups and countries. For example, in India, these worries were more strongly associated with frequent self-reported depression and anxiety among males, individuals with at least a high school education, and urban residents compared to their respective counterparts. Conversely, in Sri Lanka, we observed the opposite trend, with weaker associations in similar subgroups. These cross-country differences in effect modification are broadly consistent with the limited literature outside the pandemic context. Tran et al. [5] found that financial stress was more strongly linked to anxiety among female college students in the United States, while other studies reported no significant effect modification by gender [42-44] and similarly, no modifying role of education [42,44]. The heterogeneity in effect modification across countries underscores the limitations of one-size-fits-all approaches to mental health programming.

How do our findings compare with global research during the pandemic?

Our findings demonstrating significant associations between pandemic-related worries and poorer mental health outcomes align with previous research conducted outside South Asia during the COVID-19 pandemic [15,21,25].

In four of the five South Asian countries examined, vaccination status was associated with improved mental health outcomes—mirroring U.S. findings of reduced distress [66,67] (see S2 Table)). Intriguingly, vaccination status also emerged as an effect modifier of the relationship between pandemic-related worries and adverse mental health outcomes. Specifically, while vaccinated individuals generally reported lower levels of depression and anxiety, the strength of association between their worry and mental health

was greater than among unvaccinated individuals. One plausible explanation for this paradox is reverse causation: those who opted for vaccination may have initially had higher levels of depression or anxiety, motivating their decision to seek vaccination. Another possibility is the expectation hypothesis—vaccination may have been widely perceived as a definitive pathway back to normalcy. Consequently, vaccinated individuals might have experienced a greater sense of disappointment when pandemic disruptions continued despite vaccination. To our knowledge, this dual role of vaccination—simultaneously protective as a direct factor against depression and anxiety, yet amplifying the sensitivity of mental health outcomes to pandemic-related worries—is a novel finding.

How do our findings compare with pandemic-era studies in South Asia?

The availability of CTIS data across multiple South Asian countries over an extended period offers a rare opportunity to examine mental health outcomes within a unified study framework. Unlike meta-analyses that synthesize findings from separate country-level studies [30]—each with potentially different designs and measurement tools—our analysis benefits from a consistent methodology and survey instrument, thereby reducing heterogeneity due to study design or measurement variation.

Our findings on the significant associations between pandemic-related worries and poorer mental health in South Asia are broadly consistent with the limited existing literature in the region, which has predominantly focused on specific subgroups [37-41].

In our study, we observed that the adjusted ORs for depression and anxiety associated with pandemic-related worries varied across the five countries, with differences in the rankings of adjusted ORs among the different worry variables. These variations reflect underlying differences in pre-pandemic socio-economic structures and the coping strategies adopted by each country during the pandemic. For instance, the five countries implemented distinct lockdown policies [32], which have been shown to impact mental health in the general population [68]. Such differences in public health responses and social systems may have influenced the mental health burden in each country, highlighting the complicated interplay between policy, socio-economic resilience, and mental well-being [69].

We also noticed changes in these associations over time. Using Bangladesh as a case study, we identified a more pronounced effect of worries about food insecurity on anxiety during Period 2 (May 20, 2021, to June 25, 2022), compared to financial stress. Period 2 was marked by a sharp rise in the prices of essential commodities in Bangladesh, including food and fuel—particularly wheat and palm oil—with prices escalating further after February 2022 [70]. This surge can largely be attributed to disruptions in the global

supply chain caused by the Russia-Ukraine war [71]. These findings highlight the need for careful interpretation, as factors beyond the direct impact of COVID-19, such as geopolitical conflicts, can shape the relationship between pandemic-related stressors and mental health over time."

Conclusion:
Kindly elaborate on the study's contribution to the existing body of literature.

Response: Thank you for your comment. As noted in our previous response regarding the Introduction section, this paper makes two primary contributions to the literature. First, it is the first study to examine the association between pandemic-related worries and mental health among the general public across South Asia over an extended period (2020–2022). This represents an extension of our previous work in India (Yang et al., 2024). Prior studies in the region have typically been limited to specific countries or subpopulations (Rahman et al., 2021; Sultana et al., 2021; Lathabhavan, 2021; Lathabhavan and Vispute, 2022; Gillani et al., 2021), largely due to data constraints.

Second, our study adds to the literature by exploring the largely overlooked role of effect modifiers in shaping the association between pandemic-related worries and mental health.

We have added to "Discussion" session, on Lines 500-509, the following:

"To our knowledge, this is the first comparative study in South Asia to assess the impact of pandemic-related worries on mental health in the general population during COVID-19 from 2020 to 2022 using the same survey and deployment platform (CTIS/Meta). It extends our previous work in India [50] to a broader regional analysis and highlights the similarities and differences across South Asia. This research is particularly important given the absence of national-level monitoring systems for longitudinal tracking of mental health symptoms across countries in this region during the pandemic [74]. While most of the literature pool results from disparate studies via meta-analysis, this study uses a common global survey, reducing measurement and selection heterogeneity across countries."

We have added to "Discussion" session, on Lines 521-523, the following:

"This paper contributes to the literature by addressing the largely unexplored role of effect modifiers, including that of time and vaccination status, on the association between pandemic-related worries and mental health."

Citations and References:

This section is well organized.

General Comments:
Please remove repetition and redundancy, and improve the overall language quality. Ensure a logical flow throughout the manuscript that aligns with the objectives.

Response: Thank you for your comment. We have addressed the specific suggestions regarding the "Introduction" and "Discussion" sections. In addition, we now present the limitations and future directions in separate paragraphs to improve clarity and structure.

Reviewer 2

Thank you for addressing the feedback provided. The revisions have significantly strengthened the manuscript, and we appreciate your efforts in improving its clarity and rigor.

Response: We sincerely appreciate your thoughtful comments throughout the review process, which have greatly contributed to improving the quality of our manuscript.

Unique Citations in the Response to the Reviewers

Astley CM, Tuli G, Mc Cord KA, Cohn EL, Rader B, Varrelman TJ, et al. Global monitoring of the impact of the COVID-19 pandemic through online surveys sampled from the Facebook user base. Proceedings of the National Academy of Sciences. 2021;118(51):e2111455118.

Fang D, Thomsen MR, Nayga RM. The association between food insecurity and mental health during the COVID-19 pandemic. BMC public health. 2021;21:1–8.

Gillani S, Shafiq MN, Ahmad TI, Zaheer S. Household food insecurity and mental health amid COVID-19 Pandemic: A Case of urban informal sector labor in Punjab (Pakistan). Pakistan Journal of Social Sciences. 2021;41(4):755–772.

Hamilton HA, Wickens CM, Ialomiteanu AR, Mann RE. Debt stress, psychological distress and overall health among adults in Ontario. Journal of psychiatric research. 2019;111:89–95.

Kalin NH. The critical relationship between anxiety and depression. American Journal of Psychiatry. 2020;177(5):365–367.

Kola L, Kohrt BA, Hanlon C, Naslund JA, Sikander S, Balaji M, et al. COVID-19

mental health impact and responses in low-income and middle-income countries: reimagining global mental health. The Lancet Psychiatry. 2021;8(6):535–550.

Kundu R, Datta J, Ray D, Mishra S, Bhattacharyya R, Zimmermann L, et al. Comparative impact assessment of COVID-19 policy interventions in five South Asian countries using reported and estimated unreported death counts during 2020-2021. PLOS Global Public Health. 2023;3(12):e0002063.

Lathabhavan R. COVID-19 and mental health concerns among business owners: a cross-sectional study from India. International Journal of Mental Health and Addiction. 2023;21(6):3810–3820.

Lathabhavan R, Vispute S. Examining the mediating effects of stress on fear of COVID-19 and well-being using structural equation modeling. International Journal of Mental Health and Addiction. 2022;20(5):2681–2689

Rahman T, Hasnain MG, Islam A. Food insecurity and mental health of women during COVID-19: Evidence from a developing country. PLoS One. 2021;16(7):e0255392.

Ryu S, Fan L. The relationship between financial worries and psychological distress among US adults. Journal of family and economic issues. 2023;44(1):16–33

Sultana MS, Khan AH, Hossain S, Islam T, Hasan MT, Ahmed HU, et al. The association between financial hardship and mental health difficulties among adult wage earners during the COVID-19 pandemic in Bangladesh: findings from a cross-sectional analysis. Frontiers in psychiatry. 2021;12:635884.

Tran AG, Lam CK, Legg E. Financial stress, social supports, gender, and anxiety during college: A stress-buffering perspective. The Counseling Psychologist. 2018;46(7):846–869.

Viseu JNR, de Jesus SN, Leal ARC, Pinto PSLGdS, Ayala-Nunes L, Matavelli RD. Coping and social support as moderators: Relationship between financial threat and negative psychological outcomes. Current Psychology. 2021;40:2229–2241.

Wilson JM, Lee J, Fitzgerald HN, Oosterhoff B, Sevi B, Shook NJ. Job insecurity and financial concern during the COVID-19 pandemic are associated with worse

mental health. Journal of occupational and environmental medicine. 2020;62(9):686–691.

Wright L, Steptoe A, Fancourt D. Does thinking make it so? Differential associations between adversity worries and experiences and mental health during the COVID-19 pandemic. J Epidemiol Community Health. 2021;75(9):817–823

Yang Y, Sun A, Zimmermann L, Mukherjee B. Impact of pandemic-related worries on mental health in India from 2020 to 2022. npj Mental Health Research. 2024;3(1):57.
