# Supplementary material for: The role of financial stress, food insecurity, and COVID-19-related illness concerns shaping mental health in five South Asian countries during the pandemic (2020–2022): A secondary analysis of the online COVID-19 Trends and Impact Survey (CTIS) data
Source: PLOS Glob Public Health. 2025 Aug 8;5(8):e0004704. doi: 10.1371/journal.pgph.0004704 (PMC12334018; doi:10.1371/journal.pgph.0004704)
Supplement: Attachment — Submitted filename: PGPH-D-25-00676_reviewer_Comments.docx [file pgph.0004704.s011.docx]

**Reviewer comments**

The topic is highly relevant, and the authors present a valuable analysis. However, the manuscript titled *"The role of financial stress, food insecurity, and COVID-19-related illness concerns shaping mental health in South Asia during the pandemic years (2020–2022)"* (PGPH-D-25-00676_reviewer) requires minor revisions before it can be considered for publication.

| General comments | Kindly check the language, punctuation, and remove any redundancy. |
| --- | --- |
| Abstract | The abstract is well-written. |
| Introduction | The introduction is well-written. |
| Materials and Methods | The survey sample is not clearly defined. The manuscript should specify how the sample size was determined and provide a clear explanation of the sampling strategy. Additionally, the data collection process for the reported sample size (N = 3,644,631) needs to be described in detail, including the sources, methods used, and any inclusion or exclusion criteria applied. |
| Results | The Results section is clearly written and effectively presents the key findings. |
| Discussion | The discussion is well-written. |
| Conclusion | The conclusion is well-written. |
| References | The references appear to be well-organized. |
